# Anticoagulant Prophylaxis with Edoxaban for Asymptomatic Isolated Distal Deep Vein Thrombosis in Perioperative Hip Fracture Patients

**DOI:** 10.3400/avd.oa.26-00179

**Published:** 2026-10-27

**Authors:** Nozomu Ishikawa, Naoto Yamamoto, Tatsuro Yata, Takafumi Kayama, Yuta Yamanaka, Kazunori Inuzuka, Masaki Sano, Kazuto Katahashi, Hajime Tsuyuki, Yusuke Endo, Hiroya Takeuchi, Naoki Unno

**Affiliations:** 1 Division of Vascular Surgery, Hamamatsu Medical Center, Hamamatsu, Shizuoka, Japan; 2 Division of Vascular Surgery, Second Department of Surgery, Hamamatsu University School of Medicine, Hamamatsu, Shizuoka, Japan

**Keywords:** deep vein thrombosis, venous thromboembolism, direct oral anticoagulant (DOAC), hip fracture

## Abstract

Most distal deep vein thrombosis (DVT) presents a low risk of central progression and pulmonary thromboembolism (PTE), and uniform anticoagulation is not recommended. However, the perioperative period of hip fracture (HF) is associated with a high risk of DVT progression, but a therapeutic dose of anticoagulation raises concern about bleeding risk. There is no clear recommendation for anticoagulation for asymptomatic distal DVT during the perioperative period of HF. We retrospectively evaluated the appropriateness of general anticoagulant prophylaxis with edoxaban. From April 2017 to September 2022, among patients who underwent HF surgery at our hospital, 74 consecutive cases who were diagnosed with asymptomatic distal DVT were examined. Of the 52 cases receiving anticoagulant prophylaxis with edoxaban, 23 received the prophylactic standard dose (30 mg), and 29 received the prophylactic lower dose (15 mg). The duration of anticoagulation ranged from 5 to 28 days, with a median of 14 days. There were no recurrences of symptomatic venous thromboembolism (VTE) within 3 months after starting anticoagulation. This study suggests that a general perioperative prophylactic dose, rather than a therapeutic dose, can effectively suppress symptomatic VTE and enhance safety in patients with asymptomatic distal DVT developed during the perioperative period of HF. (This is a translation of Jpn J Phlebol 2025; 36: 33–38.)

## Introduction

Lower-extremity deep vein thrombosis (DVT) is classified as proximal DVT or isolated distal DVT according to the location of the thrombus. In the Japanese Circulation Society guidelines revised in 2017, various anticoagulants are designated as Class I recommendations for the treatment of proximal DVT.^1)^ In contrast, routine anticoagulation is not recommended for isolated distal DVT. There is also no clear recommendation regarding the management of isolated distal DVT occurring in patients with lower-extremity fractures, who are at higher risk of developing DVT than the general patient population.^1)^ In clinical practice, the type and duration of anticoagulation, as well as whether anticoagulants should be administered at all, vary among individual patients.

In Japan, anticoagulants available for the prevention of venous thromboembolism (VTE) during the perioperative period of lower-extremity orthopedic surgery include heparin, warfarin, enoxaparin, fondaparinux, and edoxaban. Among direct oral anticoagulants (DOACs), edoxaban is the only drug covered by health insurance for the prevention of VTE in patients undergoing lower-extremity orthopedic surgery, including total knee arthroplasty, total hip arthroplasty, and hip fracture (HF) surgery. Because it is administered orally and the same drug can be used for both prophylaxis and treatment, it is widely used by orthopedic surgeons. Although dose-reduction criteria for edoxaban are established according to body weight and renal function, the standard prophylactic dose of edoxaban is generally half the therapeutic dose.

We hypothesized that low-dose DOAC therapy, as an intermediate approach between therapeutic-dose anticoagulation and no anticoagulation, may be effective for asymptomatic isolated distal DVT, and we previously demonstrated that this approach may be effective and safe.^2)^ In the present study, we investigated the appropriateness of prophylaxis with edoxaban for asymptomatic isolated distal DVT during the perioperative period of HF, a condition associated with a high risk of DVT and frequently encountered in clinical practice.

## Materials and Methods

Between April 2017 and September 2022, 164 patients who underwent HF surgery at our hospital were referred to our department after DVT was detected during the perioperative period. Of these, 74 (45.1%) consecutive patients diagnosed with isolated distal DVT were included in this study. We retrospectively evaluated the anticoagulation administered based on patient characteristics and perioperative findings, as well as the subsequent outcomes.

All patients were diagnosed using lower-extremity venous ultrasonography performed in the clinical physiology laboratory at our hospital. The veins were evaluated using the compression method, and acute and chronic thrombi were distinguished based on thrombus echogenicity, compressibility, venous filling, and the appearance of mural thrombi.^3)^ The incidences of symptomatic VTE and major bleeding, as defined by the International Society on Thrombosis and Haemostasis, were evaluated from the initiation of anticoagulation until 3 months after surgery.^4)^

When edoxaban is used for prophylaxis, the standard dose is 30 mg once daily, regardless of body weight. However, a dose reduction to 15 mg once daily should be considered when the creatinine clearance (Ccr) is between 30 and 50 mL/min or when drugs with P-glycoprotein inhibitory activity are concomitantly administered. In the present study, a dose of 15 mg administered to patients meeting these dose-reduction criteria was defined as the lower dose. Patient characteristics were compared according to the edoxaban dose using the chi-square test, Fisher’s exact test, and the Mann–Whitney U test. A *p* Value <0.05 was considered statistically significant.

## Results

### Patient characteristics (**Table 1**)

**Table 1:** Clinical characteristics of perioperative patients with hip fracture with asymptomatic isolated distal deep vein thrombosis

Female sex, no. (%)	63 (85.1)
Age, y, median (range)	85.0 (77.0–89.0)
Height, cm, median (range)	149.1 (144.3–156.0)
Body weight, kg, median (range)	45.2 (38.9–51.9)
Body mass index, kg/m^2^, median (range)	20.3 (18.6–22.4)
Creatinine clearance, mL/min, median (range)	47.3 (37.0–63.9)
D-dimer (N = 67), µg/mL, median (range)	17.1 (13.2–29.1)
Antiplatelet agent, no. (%)	8 (10.8)

Of the 74 patients, 63 (85.1%) were women. The median age was 85.0 years (77.0–89.0), and the median body weight was 45.2 kg (38.9–51.9). Eight patients were receiving antiplatelet agents at the time DVT was detected because of comorbidities such as a history of cerebral infarction: aspirin in 6 patients, clopidogrel in 1, and aspirin plus cilostazol in 1. The median Ccr was 47.3 mL/min (37.0–63.9). D-dimer levels were measured in 67 patients, with a median value of 17.1 µg/mL (13.2–29.1).

### DVT

DVT was unilateral in 51 patients (25 on the right side and 26 on the left side). DVT was ipsilateral to the HF in 33 patients and contralateral in 18 patients, whereas bilateral DVT was observed in 23 patients. Ultrasonography identified acute and chronic thrombi in 21 and 53 patients, respectively.

DVT was diagnosed preoperatively in 41 patients and postoperatively in 33 patients. Among those diagnosed preoperatively, DVT was detected more than 1 day before surgery in 15 patients, on the day before surgery in 9 patients, and on the day of surgery in 17 patients. HF surgery was not postponed due to DVT.

Of the 34 patients who were diagnosed with DVT preoperatively and received anticoagulation, 31 started anticoagulation after surgery. The remaining 3 patients had all been diagnosed more than 1 day before surgery. Because early surgery was not possible due to comorbidities, anticoagulation was initiated preoperatively and withheld on the day before surgery. The durations of preoperative anticoagulation in these 3 patients were 1, 2, and 4 days, respectively. Among the patients diagnosed postoperatively, prophylaxis with edoxaban had already been initiated by orthopedic surgeons in 14 patients.

### Mechanical prophylaxis

Regardless of whether prophylactic anticoagulation was administered, mechanical prophylaxis, including compression therapy with elastic stockings or elastic bandages and intermittent pneumatic compression, was performed in all patients.

### Anticoagulation

Edoxaban was administered to 53 patients. Three patients with renal dysfunction received subcutaneous calcium heparin at a dose of 5000 units twice daily. One patient received 1.5-mg fondaparinux once daily because an orthopedic surgeon determined that oral medication was difficult because of severe dementia. Seventeen patients received no anticoagulant therapy, of whom 7 continued antiplatelet therapy (**Table 2**).

**Table 2:** Anticoagulation details of the patients

Anticoagulant agent	N = 74
Edoxaban	53
Prophylactic dose	52
Therapeutic dose	1
Subcutaneous unfractionated heparin 5000 units twice daily	3
Fondaparinux 1.5 mg once daily	1
None	17

Of the 52 patients receiving prophylaxis with edoxaban, 23 received 30 mg, and 29 received 15 mg because they met the dose-reduction criteria (**Table 3**). One patient received a therapeutic dose of edoxaban at the discretion of the orthopedic surgeon. The duration of anticoagulation ranged from 5 to 28 days, with a median of 14 days. There were no recurrences of symptomatic VTE during the 3 months after initiation of anticoagulation.

**Table 3:** Comparison of clinical characteristics in the prophylactic standard dose (edoxaban 30 mg) and the lower dose (edoxaban 15 mg)

	Prophylactic standard dose (edoxaban 30 mg) N = 23	Prophylactic lower dose (edoxaban 15 mg) N = 29	p Value
Female sex, no.	21	26	1.00
Age, y, median (range)	77.0 (73.0–82.5)	87.0 (85.5–92.0)	<0.001
Height, cm, median (range)	151.5 (148.3–158.0)	145.5 (140.0–155.0)	0.016
Body weight, kg, median (range)	46.6 (45.0–56.0)	42.1 (38.1–48.6)	0.022
Body mass index, kg/m^2^, median (range)	21.0 (19.2–22.6)	19.8 (18.6–21.4)	0.138
Creatinine clearance, mL/min, median (range)	63.2 (55.2–86.0)	39.7 (34.5–46.2)	<0.001
Thrombosis (fresh/old), no.	9/14	7/22	0.365
Major bleeding, no.	0	3	0.254
VTE recurrence, no.	0	0	1.00

Three patients receiving prophylaxis with edoxaban met the criteria for major bleeding because of a decrease in the hemoglobin level and the need for blood transfusion, accounting for 5.7% of patients receiving edoxaban. The decreases in the hemoglobin level, timing of transfusion, and amount of transfusion in these 3 patients were 4.3 g/dL, postoperative day 3, and 2 units; 1.5 g/dL, postoperative day 4, and 4 units; and 2.3 g/dL, postoperative day 6, and 2 units, respectively. In all 3 patients, the decrease in the hemoglobin level was considered to be associated with the fracture itself or the surgical procedure. No obvious internal or external bleeding was observed, and edoxaban was continued.

## Discussion

The Japanese Circulation Society guidelines revised in 2017 describe a strategy in which patients with low-risk isolated distal DVT are followed by ultrasonography after 7–21 days without anticoagulation, whereas anticoagulation is considered for high-risk patients.^1)^ High-risk patients include those with a history of VTE, patients with cancer, and patients undergoing lower-extremity orthopedic surgery. However, the guidelines state that anticoagulation may only be administered after considering the risks and benefits, and even when anticoagulation is administered, treatment for up to 3 months is considered appropriate.

The risk of DVT is reported to increase from the time a HF occurs, and several studies have reported the incidence of preoperative DVT. Although the reported incidence varies among studies, it ranges from 10.6% to 33.7%,^5–9)^ which is higher than the perioperative incidence of DVT across all clinical departments in Japan.^10,11)^ Although isolated distal DVT is considered to have a low risk of proximal extension, age ≥60 years and hospitalization are risk factors for proximal extension of isolated distal DVT,^12)^ and patients with HF often meet these criteria; all patients in the present study met both criteria. Furthermore, an analysis of fatal pulmonary thromboembolism (PTE) cases reported by the Japan Medical Safety Research Organization showed that 3 of the 8 patients for whom detailed information was available had HF.^13)^ Therefore, anticoagulation should be considered during the perioperative period in patients with HF even when DVT is isolated to the distal veins.

Management of DVT detected before HF surgery differs depending on whether the DVT is proximal or isolated distal. Prolongation of the preoperative waiting period for HF may lead to deterioration in physical function, and early surgery is recommended in the 2021 Clinical Practice Guidelines for Femoral Neck and Trochanteric Fractures.^14)^ In our department, an inferior vena cava filter is placed preoperatively in patients with proximal DVT because of the risk of embolism.

For general VTE prevention during the perioperative period of HF, guidelines in Western countries, including those of the American College of Chest Physicians (ACCP), recommend preoperative anticoagulation.^15)^ However, even in Western clinical trials, preoperative anticoagulation was performed in only 61.3% of patients, and early surgery has been reported to be 1 reason for this relatively low rate.^16)^ Preoperative anticoagulation with low-molecular-weight heparin has also been reported not to significantly reduce the risk of postoperative VTE compared with postoperative initiation.^17,18)^

In addition, preoperative anticoagulation can be difficult in patients with HF because bleeding from the fracture site may exacerbate anemia. Therefore, even when isolated distal DVT is present, there is no need to delay HF surgery to administer preoperative anticoagulation, and postoperative anticoagulation for 10–14 days, as recommended with a Grade 1B recommendation in the ACCP guidelines, may be sufficient.^15)^ However, intra-articular bleeding has been reported after administration of a standard-dose DOAC for isolated distal DVT after orthopedic surgery.^19)^ Furthermore, in a study of patients undergoing lower-extremity orthopedic surgery, the incidence of hemorrhagic adverse events tended to increase as Ccr decreased.^20)^ In the present study, the median Ccr was below 50 mL/min, the median body weight was 45.2 kg, and all patients weighed <60 kg. Therefore, the possibility of hemorrhagic adverse events should be carefully considered when initiating anticoagulation.

The standard prophylactic dose of edoxaban is 30 mg; however, a dose reduction to 15 mg (lower dose) should be considered in patients with a Ccr between 30 and 50 mL/min. The decision to administer the standard prophylactic dose or a lower dose should be based on the bleeding risk associated with the characteristics of each patient. In the present study, patients who met the dose-reduction criteria tended to be older, have a lower body weight, and have impaired renal function.

According to the guidelines, the duration of anticoagulation for DVT is 3 months when the risk factor is reversible, and treatment for up to 3 months is considered appropriate for isolated distal DVT even when anticoagulation is administered. In contrast, the duration of prophylaxis with edoxaban is 10–14 days.^1)^ Based on these considerations, 4 possible patterns of edoxaban administration in patients with HF and isolated distal DVT can be considered: (1) administration of a therapeutic dose of edoxaban for 3 months; (2) administration of edoxaban at a dose lower than the therapeutic dose for 3 months, that is, treatment of DVT with a lower dose of edoxaban; (3) prophylaxis with edoxaban for 10–14 days, that is, use of edoxaban for prophylactic purposes; and (4) no administration of edoxaban, that is, no anticoagulation.

Pattern 1 raises concerns regarding adverse hemorrhagic events. Patterns 2 and 3 are similar in that the dose of edoxaban is lower than the therapeutic dose; however, the duration of administration differs depending on whether the purpose is treatment or prophylaxis. Pattern 4 raises the question of whether clinically significant symptomatic PTE can be prevented; in this situation, mechanical prophylaxis with compression therapy or intermittent pneumatic compression would be used. HF is associated with both the bleeding risk and the risk of proximal thrombus extension. However, considering that patients are generally able to ambulate postoperatively, we considered Pattern 3, in which a DOAC at a dose lower than the therapeutic dose is administered for 10–14 days to prevent proximal thrombus extension, to be reasonable when DVT is isolated to the distal veins.

Among the patients who received 15 mg of edoxaban in the present study, 3 had decreases in hemoglobin levels that met the criteria for major bleeding and required blood transfusion. One patient, whose hemoglobin level decreased by 4.3 g/dL and who received 2 units of blood on postoperative day 3, had received anticoagulation for 1 day preoperatively. The proportion of patients who received blood transfusions was 5.7% among patients treated with edoxaban and 4.1% in the overall study population.

The small sample size precluded the determination of the extent to which prophylaxis with edoxaban, including preoperative administration, contributed to these events. This represents a limitation of the present study, and further investigation in a larger cohort is required. However, several studies of blood transfusion in patients with HF have reported that approximately 14.6%–49.4% of patients required postoperative transfusion.^21–24)^

In addition, a comparison between patients receiving and not receiving DOACs for isolated distal DVT associated with orthopedic conditions revealed that major bleeding occurred only in the DOAC group, although the difference was not statistically significant. Omura et al. concluded that DOACs may be an option if thrombotic and bleeding risks are carefully assessed because the causes of bleeding events were mainly related to the underlying orthopedic conditions.^25)^ In the present study, the need for blood transfusion was also considered more likely to be attributable to the expected clinical course of the fracture or surgical procedure than to a direct effect of anticoagulation, and edoxaban could therefore be continued.

However, in perioperative patients with HF who are considered to be at high risk of bleeding and are also likely to require postoperative transfusion, DOACs should be administered cautiously even at a lower dose. In fact, no symptomatic VTE occurred in any patient, including the 17 patients who did not receive anticoagulation after comprehensive consideration of thrombus characteristics, such as a chronic or small thrombus, and bleeding risk; 7 of these patients continued the antiplatelet agents they had already been taking.

Therefore, routine anticoagulation solely because of excessive concern about the development of PTE should be avoided. Management should be individualized according to each patient, including mechanical prophylaxis with delayed initiation of a DOAC until anemia improves or mechanical prophylaxis without pharmacological anticoagulation.

## Conclusion

Although the number of patients was limited and further investigation is required, this retrospective study suggests that asymptomatic isolated distal DVT occurring during the perioperative period of HF does not require specific treatment, such as therapeutic-dose anticoagulation, and that routine mechanical and pharmacological prophylaxis used during the perioperative period of HF may safely prevent the development of symptomatic VTE.
